# AoPrdx2 Regulates Oxidative Stress, Reactive Oxygen Species, Trap Formation, and Secondary Metabolism in *Arthrobotrys oligospora*

**DOI:** 10.3390/jof10020110

**Published:** 2024-01-28

**Authors:** Na Zhao, Meichen Zhu, Qianqian Liu, Yanmei Shen, Shipeng Duan, Lirong Zhu, Jinkui Yang

**Affiliations:** State Key Laboratory for Conservation and Utilization of Bio-Resources, Key Laboratory for Microbial Resources of the Ministry of Education, School of Life Sciences, Yunnan University, Kunming 650091, China; zn0314@mail.ynu.edu.cn (N.Z.); zmc201789@163.com (M.Z.); liuqianqian614@163.com (Q.L.); shenyanmei@stu.ynu.edu.cn (Y.S.); duanshipeng@stu.ynu.edu.cn (S.D.); zhulirong@stu.ynu.edu.cn (L.Z.)

**Keywords:** peroxiredoxin, gene disruption, stress response, pathogenicity, secondary metabolism

## Abstract

Prdx2 is a peroxiredoxin (Prx) family protein that protects cells from attack via reactive oxygen species (ROS), and it has an important role in improving the resistance and scavenging capacity of ROS in fungi. *Arthrobotrys oligospora* is a widespread nematode-trapping fungus that can produce three-dimensional nets to capture and kill nematodes. In this study, AoPrdx2, a homologous protein of Prx5, was investigated in *A. oligospora* via gene disruption, phenotypic analysis, and metabolomics. The deletion of *Aoprdx2* resulted in an increase in the number of mycelial septa and a reduction in the number of nuclei and spore yield. Meanwhile, the absence of *Aoprdx2* increased sensitivity to oxidative stresses, whereas the ∆*Aoprdx2* mutant strain resulted in higher ROS levels than that of the wild-type (WT) strain. In particular, the inactivation of *Aoprdx2* severely influenced trap formation and pathogenicity; the number of traps produced by the ∆*Aoprdx2* mutant strain was remarkably reduced and the number of mycelial rings of traps in the ∆*Aoprdx2* mutant strain was less than that of the WT strain. In addition, the abundance of metabolites in the ∆*Aoprdx2* mutant strain was significantly downregulated compared with the WT strain. These results indicate that AoPrdx2 plays an indispensable role in the scavenging of ROS, trap morphogenesis, and secondary metabolism.

## 1. Introduction

Plant pathogenic nematodes are widely distributed, comprising hundreds of species [1]. In China, nematodes cause serious damage to almost all cash crops, such as wheat, soybeans, and vegetables, and threaten the safety of food and cash crops [2]. People have long been aware of the harm caused by plant pathogenic nematodes, and researchers have been searching for ways to control nematodes since the 1940s [3]. Over the past few decades, the control of plant pathogenic nematodes has been mainly based on chemical methods, but the damage to the environment and plants caused by these chemicals is irreversible [4]. In 1920, Cobb in the United States proposed the biological control of plant parasitic nematodes. Subsequently, scientists from various countries have made great efforts, and many biocontrol factors have been discovered successively, but research on these biocontrol factors mainly focuses on nematophagous fungi [5]. Nematode-trapping (NT) fungi are a major category of nematophagous fungi, which can capture, colonize, and digest nematodes by forming trapping devices (traps) [6]. Their interactions with nematodes have led to the evolution of complex mycelial traps, such as adhesive nets, adhesive knobs, and constricting rings [7]. Among them, *Arthrobotrys oligospora*, a widespread NT fungus, preys on nematodes by forming adhesive nets [8]. *A. oligospora* is a typical species for studying the interaction between fungi and nematodes [9]. In recent years, it has been confirmed that there are several important genes and cellular processes involved in the trap formation of *A. oligospora*, including peroxisome-related genes [10], G protein signaling [11], the mitogen-activated protein kinase signaling pathway [12], and sporulation-related genes [13].

The chemical properties of reactive oxygen species (ROS) are very active, and a large amount of ROS will attack the cells, amino acids, fluid lipids, etc., resulting in toxicity and cell damage [14]. In addition, ROS participate in various intracellular signaling pathways, including p53 and NF-xP, to regulate metabolic processes [15]. Accordingly, intracellular antioxidants of various types are available to resist the toxic effects of ROS on cells, such as superoxide dismutase, peroxidase, glutaroglycin, and vitamins [16,17]. Peroxiredoxin (Prx) is an antioxidant protein that can decompose peroxide and belongs to the peroxide reductase family of proteins [18]. Prx is one of the three mechanisms for decomposing hydrogen peroxide (H_2_O_2_) to prevent cells from being attacked by ROS, and includes peroxide and alkyl hydroperoxide. Prx was first discovered in *Saccharomyces cerevisiae* [19] and was later reported in bacteria, archaea, and other eukaryotes [20]. Therefore, Prx is a widely distributed enzyme with high expression levels, and it is one of the top ten proteins with the highest content in *Escherichia coli* [21]. H_2_O_2_ is recognized as the major ROS in the redox regulation of biological activities, and Prxs catalyze the removal of H_2_O_2_. Prx is mainly located in the cytoplasm, and almost all H_2_O_2_ in the cytoplasm is reduced by Prx. Prx decomposes H_2_O_2_ into H_2_O and O_2_ in its catalytic reaction and reduces H_2_O_2_ to H_2_O by oxidizing hydrogen donor compounds in its peroxidation reaction [22]. The decomposition of the hydroperoxide catalyzed by Prx depends on its own oxidation–reduction activity involving two cysteines, namely peroxidaticcysteine and resolving cysteine [23]. Indeed, numerous members of the Prx superfamily have been identified and characterized in prokaryotes, archaea, and eukaryotes. Prx can be classified into six subfamilies, namely, AhpC/Prx1, Prx6, Prx5, Tpx, BCP/PrxQ, and AhpE [24,25].

In recent years, Prx family proteins have been extensively documented for their important functions in several fungi. In yeast, the peroxide-reducing protein Tsa1 protects yeast cells from toxic levels of DNA damage occurring during aerobic growth, promotes resistance to H_2_O_2_, and prolongs the cellular lifespan under heat limitation when ROS levels are elevated as a result of oxidative stress induced by heat stress injury [26,27,28]. Tpx1, the major peroxide-reducing protein in fission yeast, is important for maintaining aerobic growth and contributes significantly to cellular defense against oxidative damage [29]. Typical 2-Cys Prxs in the model organisms *Schizosaccharomyces pombe* and *S. cerevisiae* also have roles in regulating signaling, the DNA damage response, and as molecular chaperones [30]. In addition, Prx plays a role in the antioxidant defense mechanisms of fungi in *Aspergillus nidulans*, *Paracoccidioides brasiliensis*, *Candida glabrata*, and *Aspergillus fumigatus* [31,32,33,34]. Prx is also required for virulence, and in *A. nidulans*, *C. glabrata*, *A. fumigatus*, and *Fusarium graminearum*, Prx is known to enhance the mediation of their lethal effects [31,33,34,35]. In *A. nidulans*, Prx is also involved in the regulation of conidial specificity [31]. Therefore, Prx plays an irreplaceable role in defense against oxidative stress, virulence regulation, spore development, signal transduction, and prolongation of the cellular lifespan in fungi [36].

Recently, an NADPH oxidase AoNoxA was identified in *A. oligospora*, which is involved in ROS synthesis. The inactivation of *AonoxA* resulted in a dramatic reduction in ROS levels and trap formation induced by the nematode *Caenorhabditis elegans* [37]. However, little is known about the roles and related mechanisms of ROS synthesis and decomposition in NT fungi. Here, we characterized a homologous Prx5 (AoPrdx2) in *A. oligospora* via phenotypic comparison and metabolome analyses.

## 2. Materials and Methods

### 2.1. Organisms and Media

The wild-type (WT) fungus *A. oligospora* (ATCC24927) was purchased from the American Type Culture Collection (ATCC) (Manassas, VA, USA) and the derived knockout strains (∆*Aoprdx2* mutant strains) were cultured in potato dextrose agar (PDA) medium at 28 °C. The *S. cerevisiae* strain (FY834) was incubated in yeast extract peptone dextrose (YPD) medium as a host for constructing recombinant plasmids [38]. The *E. coli* strain (DH5α) was incubated in a lysogeny broth medium and was used as a host to preserve plasmids pRS426 (cloning vector) and pCSN44 (containing the hygromycin resistance gene *hph*). In addition, PDA, tryptone glucose (TG), and tryptone yeast extract glucose agar (TYGA) were used to compare the fungal phenotypic traits, as described previously [39]. The *C. elegans* (strain N2) was cultured in oat medium at 26 °C to induce trap formation.

### 2.2. Sequence Analysis of AoPrdx2

AoPrdx2 (AOL_s00043g804) was retrieved from the genome of *A. oligospora* based on the homologs from the model fungus *Aspergillus nidulans* (Q5AXN8) and *Neurospora crassa* (XP_964200). The partial properties of AoPrdx2 were analyzed using the pI/MW tool (http://www.expasy.ch/tools/pi_tool.html) (accessed 14 March 2023). The homologs of Prdx2 from various fungi were searched for and downloaded from GenBank. Their sequence similarity was analyzed using DNAman (version 5.22), and Mega (version 7.0) was used to construct the neighbor-joining tree [40].

### 2.3. Deletion of Aoprdx2

The deletion of *Aoprdx2* was performed using the homologous recombination method [41,42]. First, the upstream and downstream homologous arms of the target gene were amplified from the genomic DNA of *A. oligospora*, and *hph* was selected as a screening marker, which was amplified from the pCSN44 plasmid with paired primers (Table S1). The pRS426 plasmid was digested with *EcoRI* and *XhoI*, and the linearized pRS426 and the amplified fragments were co-transferred to *S. cerevisiae* (FY834) via electroporation [43]. Then, the constructed recombinant plasmid PRS426-AoPrdx2-hph was transferred into *A. oligospora* using protoplast transformation, as described previously [43,44]. The transformants were selected on PDAS (PDA supplemented with 10 g/L molasses and 0.4 M saccharose) medium containing 200 µg/mL of hygromycin B (Amresco, Solon, OH, USA) [45]. Finally, these transformants were further verified using PCR and Southern blotting analyses [43].

### 2.4. Comparison of Mycelial Growth and Sporulation

The WT and knockout strains were cultured in PDA, TYGA, and TG plates at 28 °C for 5 days, and the colony diameters were measured every day [44]. The mycelia of the WT and knockout strains were stained with 20 μg/mL of cell-wall-specific calcium fluorescent white (CFW, Sigma-Aldrich, St. Louis, MO, USA) or nuclear-specific 4′,6′-diamino-2-phenylindole (DAPI, Sigma-Aldrich, USA) for 15 min to observe the septa and nuclei of the mycelia. Mycelium morphology and the number of nuclei were observed using an inverted fluorescence microscope [46,47].

The WT and knockout strains were inoculated into a triangular flask containing 60 mL of corn meal yeast extract (CMY) medium. After incubation at 28 °C for 14 days, 20 mL of sterile water was added to wash the spores, and the conidia yield was determined as previously described [48]. Then, 50 μL of conidial suspension (2 × 10^4^ spores) was incubated in Vogel’s minimal medium (MM, 20 mL/L Vogel’s salts and 15 g/L sucrose) at 28 °C, and the spore germination rates were determined at 4, 8, and 12 h. The fresh spores were stained with CFW, and the spore morphology was recorded with photographs [49].

### 2.5. Analysis of Stress Response

The WT and knockout mutants were inoculated in TG medium containing different concentrations of stressed reagents. Different concentrations of sorbitol (0.25–0.75 M) and NaCl (0.1–0.3 M) were used as osmotic stress reagents, SDS (0.01–0.03%) and Congo red (30–90 µg/mL) were used as cell-wall-disturbing reagents, and H_2_O_2_ (5–15 mM) and menadione (0.05–0.09 mM) were used as oxidative stress reagents [11]. After incubation at 28 °C for 6 days, the colony diameter of the WT and mutant strains was determined, and the relative growth inhibition rate (RGI) was calculated as previously described [50].

### 2.6. Analysis of Trap Formation and Nematode Predation Efficiency

An amount of 50 μL of conidial suspension (2 × 10^4^) was incubated in a water agar (WA) plate and cultured at 28° for 3 days. Then, about 400 nematode *C. elegans* N2 were added per plate to induce trap formation. After induction for 12, 24, 36, and 48 h, the number of traps and captured nematodes were observed, counted, and photographed [51]. In addition, the trap morphology was observed using CFW staining [52].

### 2.7. Analysis of ROS Level and Endocytosis

To detect the ROS level, the WT and mutant strains were stained with 10 µg/mL of dihydroethidium (DHE) (MCE, Shanghai, China) and were observed under a fluorescence microscope after staining for 30 min. Photographs were taken and the fluorescence intensity was calculated using Image J [53]. In addition, the fungal strains were cultured in PDA plates for 3 days, and 20 mL of 0.2% nitrotetrazolium blue chloride (NBT) (Solarbio, Beijing, China) solution was used for staining in the dark. After dyeing at 28 °C for 30 min, the supernatant was discharged, the sample was rinsed twice with ethanol, and the plate was re-incubated in the dark at 28 °C for 30 min before imaging [54]. In addition, in order to compare the differences in endocytosis between the WT and mutant strains, the fresh mycelia samples were stained with FM4-64 (MCE, Shanghai, China), and the entry of dye into mycelia at different time points was recorded from 0 min [54].

### 2.8. Analysis of the Metabolites

The WT and knockout strains were cultured in potato dextrose (PD) broth at 28° for 6 days. Then, the fermentation broth was harvested via filtration, mixed with an equal volume of ethyl acetate, ultrasonicated three times (20 min each time), and then dried using a vacuum rotary evaporator. The crude samples were dissolved in 1 mL of methanol and dried at room temperature to obtain the dry weight of the samples. The final concentration of each sample was adjusted to 10 mg/mL through the addition of methanol (Table S2), and then the samples were filtered three times through a 0.22 μm organic phase filter. The samples were then loaded into a running sample vial and the program was set up in an ultra-performance liquid chromatography–tandem mass spectrometry (UPLC-MS/MS) instrument before the sample was run. LC-MS analysis was performed later and analyzed as described previously [55].

### 2.9. Statistical Analysis

All experiments were performed with three repetitions, and the data are represented as mean ± standard deviation (SD). Prism 8.0 (GraphPad Software, San Diego, CA, USA) was used for one-way analysis of variance. In this experiment, *p* < 0.05 was considered significant.

## 3. Results

### 3.1. AoPrdx2 Sequence and Phylogenetic Analysis

*Aoprdx2* encodes a protein composed of 169 amino acid residues with a theoretical molecular weight of 18.3 kDa and a pI of 5.1. AoPrdx2 contains a conserved PRX5-like domain. AoPrdx2 has high sequence similarities (76.6–92.4%) with homologs of three other NT fungi, and 56.1–63.7% similarity with homologs of other fungi, such as *A. nidulans* (63.7%) and *N. crassa* (56.7%). A phylogenetic tree of the AoPrdx2 homologous proteins from different fungi was constructed, and NT fungal homologous AoPrdx2 proteins were divided into a single clade (Figure S1).

### 3.2. Verification of Knockout Strains

The encoding gene of *Aoprdx2* was replaced with an *hph* fragment, and transformants were selected in a PDAS plate containing hygromycin (Figure S2A,B). After isolation, the whole genome DNA of the transformants was extracted, and the positive transformants were validated via PCR amplification using primers yz-5f and yz-3r (Table S1). The fragment sizes of the WT and transformant were 2440 and 3351 bp, respectively (Figure S2C). Then, three positive mutants (∆*Aoprdx2-1*, ∆*Aoprdx2-2*, and ∆*Aoprdx2-3*) were verified using Southern blotting analysis (Figure S2D).

### 3.3. AoPrdx2 Impairs the Development of Mycelial Septa and Nuclei

**Figure 1:**
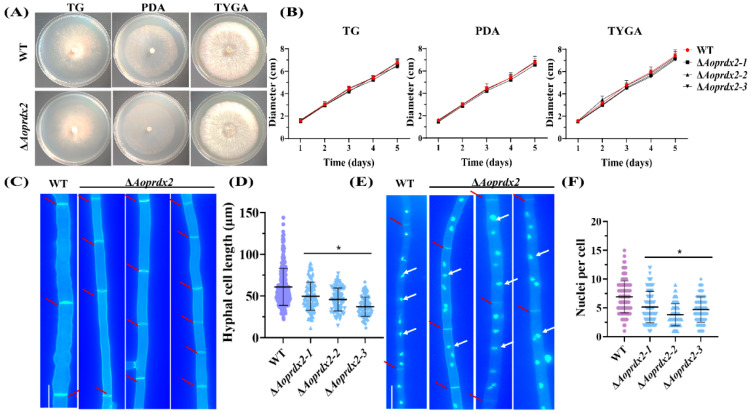
Comparison of the mycelial growth, the cell length, and the number of nuclei in the wild-type (WT) and ∆*Aoprdx2* mutant strains. (**A**) Colony morphology of the WT and ∆*Aoprdx2* mutant strains cultured in different media at 28 °C for 5 days. (**B**) Comparison of the colony diameters in three media. (**C**) Hyphal morphology of the WT and ∆*Aoprdx2* mutant strains after calcium fluorescent white (CFW) staining. Scale bar: 10 μm. The red arrows indicate the hyphal septa. (**D**) Mycelial cell length differences between the WT and ∆*Aoprdx2* mutant strains. Error bar: standard deviation from 100 replicates. (**E**) The nucleus within each cell after staining with CFW and 4′,6′-diamino-2-phenylindole for the WT and ∆*Aoprdx2* mutant strains. Scale bar: 10 μm. The red arrows indicate the hyphal septa and the white arrows indicate the nuclei. (**F**) Comparison of the number of nuclei in a single cell between the WT and ∆*Aoprdx2* mutant strains. Error bar: standard deviation from 100 replicates. (**D**,**F**) * Indicates that the ∆*Aoprdx2* mutant strain is significantly different from the WT strain (Tukey’s HSD, *p* < 0.05).

When the WT and the ∆*Aoprdx2* mutant strains were cultured for 5 days in TG, PDA, and TYGA media, respectively, there was no difference in the mycelial growth between them (Figure 1A,B). However, the number of septa significantly increased in the ∆*Aoprdx2* mutant strain after CFW staining, which resulted in a reduction in the hyphal cell length (13.32–29.18 μm) of the ∆*Aoprdx2* mutant strain compared with the WT strain (38.83–83.35 μm) (Figure 1C,D). Moreover, after staining with DAPI, the number of nuclei in the ∆*Aoprdx2* mutant strain ranged from 2–7, which was less than that in the WT strain (4–10 nuclei per cell) (Figure 1E,F).

### 3.4. AoPrdx2 Regulates Tolerance to External Stresses

**Figure 2:**
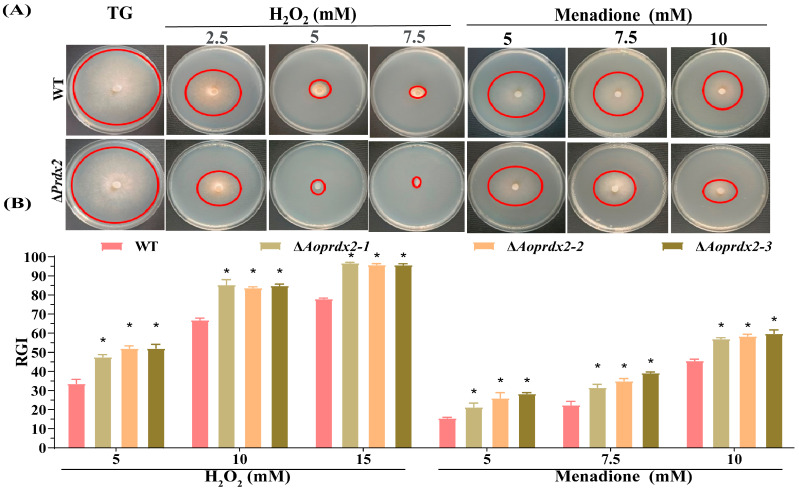
Comparison of the stress response to oxidative stress reagents of the wild-type (WT) strain and ∆*Aoprdx2* mutants. (**A**) Colony morphology of the WT and ∆*Aoprdx2* mutant strains in tryptone-glucose (TG) medium with different concentration gradients of H_2_O_2_ and menadione. The red circle indicates the edge of the colony. (**B**) Comparison of the relative growth inhibition rates (RGIs) corresponding to the WT and ∆*Aoprdx2* mutant strains. * Indicates that the ∆*Aoprdx2* mutant strain is significantly different from the WT strain (Tukey’s HSD, *p* < 0.05).

The test of resistance to external stress included multiple stresses from oxidants, osmotic reagents, and cell-wall-disturbing reagents. Compared with the WT strain, the growth of the ∆*Aoprdx2* mutant strains was inhibited by oxidative stress reagents H_2_O_2_ (2.5, 5, and 7.5 mM) and menadione (5, 7.5, and 10 mM) with different concentration gradients, and the RGI values were remarkably increased (Figure 2A,B). In contrast, Congo red (0.03–0.09 mg/mL) contributed to the mycelial growth of the ∆*Aoprdx2* mutant strain, and the RGI values were obviously reduced in the ∆*Aoprdx2* mutant strain compared to the WT strain, whereas the SDS and hypertonic stress reagents (NaCl and sorbitol) had no influence on the mycelial growth of the ∆*Aoprdx2* mutant strain (Figure S3).

### 3.5. AoPrdx2 Regulates Sporulation

**Figure 3:**
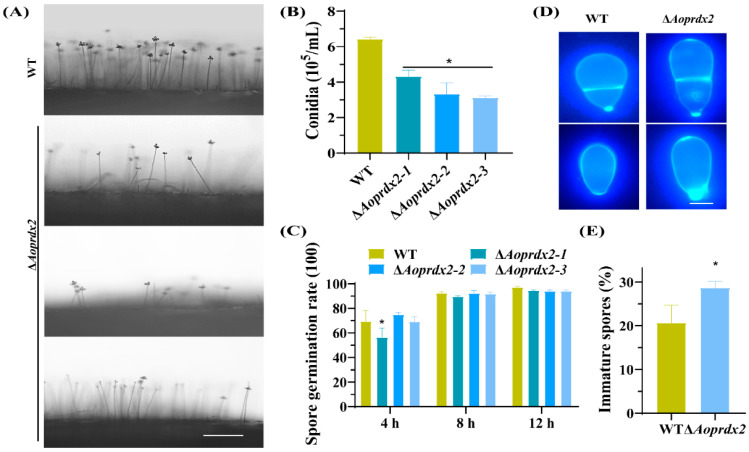
The effects of AoPrdx2 on the sporulation of the wild-type (WT) strain and ∆*Aoprdx2* mutants. (**A**) Microscopically photographed conidiophores of the WT and ∆*Aoprdx2* mutant strains in PDA media. Scale bar: 50 µm. (**B**) Spore yields 15 days post-incubation in corn meal yeast extract medium. (**C**) The germination rates of spores at different time points in minimal medium. (**D**) Mature and immature spore morphology under calcium fluorescent white staining. Scale bar: 10 µm. (**E**) Comparison of immature spores. (**B**,**E**) * Indicates that the ∆*Aoprdx2* mutant strain is significantly different from the WT strain (Tukey’s HSD, *p* < 0.05).

Using the side-shot method, the conidiophores were observed in the WA plate, and the number of conidiophores in the ∆*Aoprdx2* mutant strain was remarkably lower than that of the WT strain (Figure 3A), which is consistent with the statistical data on the conidia yields of the WT (6.4 × 10^5^ spores per mL) and ∆*Aoprdx2* mutant strains (3.0 × 10^5^ spores per mL) (Figure 3B). There was no significant difference in the spore germination rate of the WT and ∆*Aoprdx2* mutant strains (Figure 3C). In addition, the fresh conidia were stained with CFW. Mature conidia have a septum, whereas unmatured spores lack a septum. Compared with the WT strain, the spores of the ∆*Aoprdx2* mutant strains became longer and more spores were immature (Figure 3D,E).

### 3.6. AoPrdx2 Regulates Trap Formation and Nematode Predation Efficiency

**Figure 4:**
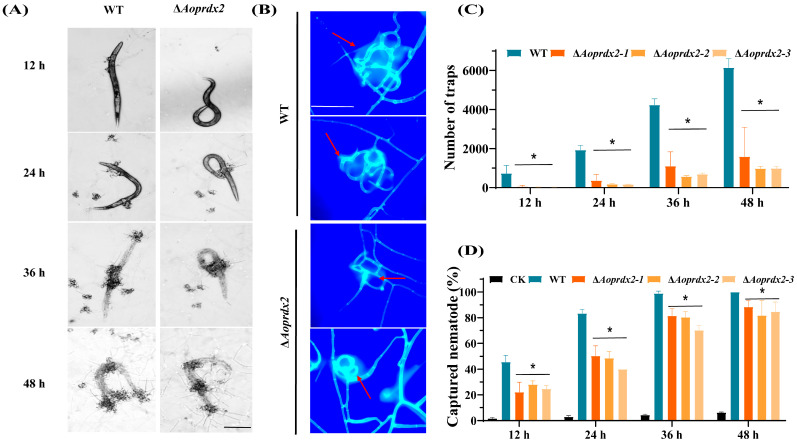
The effects of AoPrdx2 on trap formation and nematocidal activity of the wild-type (WT) strain and ∆*Aoprdx2* mutants. (**A**) Images of traps produced by the WT and ∆*Aoprdx2* mutant strains at different times. Scale bar: 100 µm. (**B**) Microscope image of the traps at 48 h. Scale bar: 10 µm. The red arrows indicate the traps. (**C**) Statistics of the number of traps at different time points. (**D**) Comparison of captured nematodes. (**C**,**D**) * Indicates that the ∆*Aoprdx2* mutant strain is significantly different from the WT strain (Tukey’s HSD, *p* < 0.05).

Nematode predation is one of the important biological functions of NT fungi. After being induced with nematode *C. elegans* N2, traps were formed in the plates containing the WT strain, whereas the number of traps was significantly reduced in the plates containing the ∆*Aoprdx2* mutant strain at different induction times (Figure 4A,B). Accordingly, the nematode predation efficiency of the ∆*Aoprdx2* mutant was remarkably decreased compared to that of the WT strain (Figure 4C). After staining with CFW, it could be seen that the traps of the WT strain contained more mycelial rings (5–7 rings) than the ∆*Aoprdx2* mutant (2’3 rings) (Figure 4D).

### 3.7. AoPrdx2 Regulates ROS Accumulation and Endocytosis

**Figure 5:**
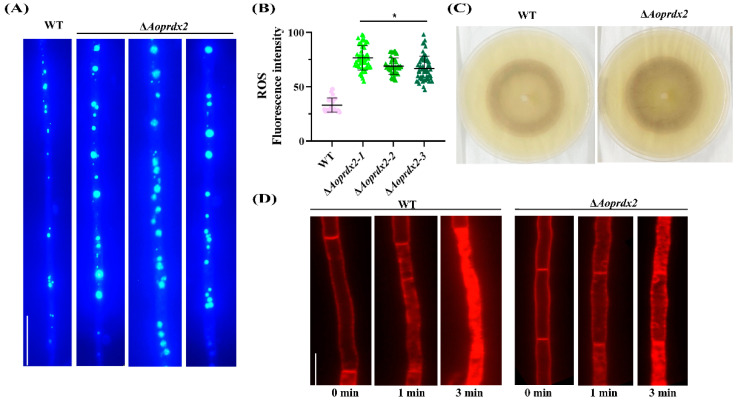
Analysis of AoPrdx2 in ROS accumulation and endocytosis in the wild-type (WT) strain and ∆*Aoprdx2* mutants. (**A**) Comparison of dihydroethidine staining. Scale bar: 10 µm. (**B**) Comparison of ROS fluorescence intensity. Error bar: standard deviation from 100 replicates. * Indicates that the ∆*Aoprdx2* mutant strain is significantly different from the WT strain (Tukey’s HSD, *p* < 0.05). (**C**) Comparison of nitrotetrazolium blue staining imaging. (**D**) The fresh mycelia were stained with FM4-64 at different time points. Scale bar: 10 µm.

DHE is one of the most commonly used superoxide anion fluorescent probes, which can effectively detect ROS levels [56]. After staining with DHE, the mycelia of the ∆*Aoprdx2* mutant accumulated more ROS compared with the WT strain (Figure 5A). After obtaining the statistics on fluorescence intensity, it was found that the fluorescence intensity of the WT and ∆*Aoprdx2* mutant strains was 67.31–87.77 and 15.35–21.80, respectively (Figure 5B). Accordingly, the mycelia of the ∆*Aoprdx2* mutant were more strongly stained by NBT than those of the WT strain, all of which indicated a greater accumulation of ROS in the ∆*Aoprdx2* mutant strain (Figure 5C). In the endocytosis analysis, it was observed that the WT mycelia almost completely entered the cytoplasm and vacuole after being co-incubated with FM4-64 dye solution for 3 min. In contrast, the mycelia of mutant Δ*Aoprdx2* did not fully internalize the FM4-64 dye solution after 3 min (Figure 5D).

### 3.8. AoPrdx2 Impairs Secondary Metabolism

**Figure 6:**
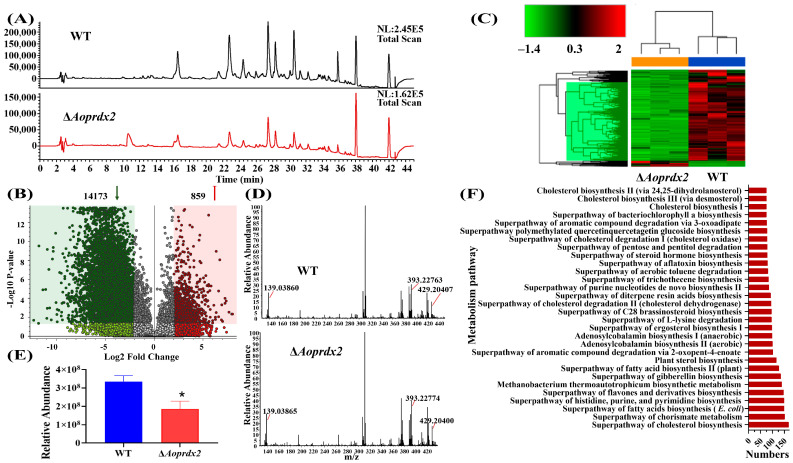
Comparison of the metabolome of the wild-type (WT) strain and ∆*Aoprdx2* mutant. (**A**) Chromatographic comparison. (**B**) Comparison of upregulated and downregulated compounds via volcano plot analysis. (**C**) Heatmap for upregulated and downregulated metabolic pathways. (**D**) Diagnostic fragment ion peaks of arthrobotrisins. (**E**) Comparison of peak areas of arthrobotrisins. (**F**) Top 30 pathways associated with differentially expressed compounds. * Indicates that the ∆*Aoprdx2* mutant strain is significantly different from the WT strain (Tukey’s HSD, *p* < 0.05).

After 6 days of incubation in PD broth, the crude extracts were obtained via extraction with ethyl acetate, and appropriate amounts of methanol were added according to the dry biomass of the WT and ∆*Aoprdx2* mutant strains to achieve a final concentration of 10 mg/mL for both. The LC-MS analysis revealed differences in the abundance of compounds between the WT and ∆*Aoprdx2* mutant strains. Comparing the chromatogram peak values of the WT and ∆*Aoprdx2* mutant strains, the abundance of metabolites in the ∆*Aoprdx2* mutant was decreased, with much lower peaks at 16–36 min (Figure 6A). The volcano plot analysis showed that 14,173 compounds were downregulated and 859 compounds were upregulated in the ∆*Aoprdx2* mutant strain (Figure 6B). The corresponding clustered heatmaps showed a large difference in the metabolic profiles of the WT and ∆*Aoprdx2* mutant strains, with most of the metabolic pathways downregulated in the ∆*Aoprdx2* mutant strain (Figure 6C). In addition, specific metabolite arthrobotrisins (diagnostic fragments at ions 139.03, 393.33, and 429.20 *m*/*z* under negative ion conditions) [51] were found in both the WT and ∆*Aoprdx2* mutant strains (Figure 6D), and the peak areas of arthrobotrisins were remarkably downregulated in the ∆*Aoprdx2* mutant strain (Figure 6E). The most enriched differential metabolic pathways were related to the biosynthesis of cholesterol and fatty acids (Figure 6F).

## 4. Discussion

AoPrdx2, a member of the Prx family of proteins, also known as thioredoxin peroxidases (Tpx) or “protective proteins”, is an important conserved protein involved in antioxidant defense and redox signaling. It is able to regulate signaling cascades due to their antioxidant and chaperone functions [20,57]. Homologous proteins of AoPrdx2 are found in various filamentous fungi, and they share a high degree of similarity in their sequences. Here, we identified the function of AoPrdx2 in a typical NT fungus, *A. oligospora*, including its role in oxidative stress responses, ROS accumulation, sporulation, and trap formation.

The deletion of *Aoprdx2* has no influence on mycelial growth but causes obvious effects on mycelial septa and nuclei. The mycelia of the ∆*Aoprdx2* mutant strains contain more septa, which resulted in shortened cell length compared with the WT strain. The mycelia of the WT strain contain more nuclei than the ∆*Aoprdx2* mutant strain. In addition, the inactivation of *Aoprdx2* impaired spore development, resulting in a reduction in spore production (about 50%) and a variable rate of spore maturation. Similar phenomena can be observed in *A. nidulans*, with a reduction in asexual spore production of about 54% in the ∆*prxA* mutant and a lesser reduction in conidial spore production in the ∆*prxB* mutant (about 20%) [31]. These results suggest that the homologs of AoPrdx2 play a crucial role in mycelia development and sporulation.

In many fungal pathogens, Prx homologs serve as virulence factors and are involved in ROS scavenging [33,58]. In *Candida albicans*, CaTsa1 is required for the yeast–hyphae transition under oxidative stress [59], and oxidized Tsa1p is greatly increased in hyphal cells, indicating its active function in the pathogenic state, where greater levels of ROS are produced [60]. Prx protein Asp f3 is required for *A. fumigatus* virulence in experimental pulmonary aspergillosis [58]. In addition, knockout neutrophil survival tests for Tsa1 and Tsa2 were performed in *C. glabrata* to determine their virulence, and the results showed that strains lacking Tsa1 and Tsa2 exhibited significantly reduced survival rates, less than 50% compared to their parental strains. Both Tsa1 and Tsa2 are essential for neutrophilic survival and for the virulence of *C. glabrata* [33]. Here, our results showed that the deletion of *Aoprdx2* had a severe impact on trap development. The number of traps was remarkably reduced in the ∆*Aoprdx2* mutant, and the number of mycelial rings of traps in the ∆*Aoprdx2* mutant was also less than that of the WT strain. Accordingly, the nematode predation efficiency of the ∆*Aoprdx2* mutant was remarkably impaired. These results showed that Prx homologs play a conserved role in different pathogens, and AoPrdx2 is critical for trap formation and the morphological development of *A. oligospora*.

Prx homologs have been proven to be involved in antioxidant defense and redox signaling [20,57]. In *A. nidulans*, Δ*prxA* showed high sensitivity to H_2_O_2_ and menaquinone [31]. In *A. fumigatus*, Δ*prx1* was more sensitive than the WT strain to stress conditions, such as menaquinone [34]. NAPDH oxidation was evaluated via colorimetry to determine Prx activity indirectly, and the substitution of Asp f3 with a serine residue reduced the peroxidase activity of Asp f3. The effects of extracellular O^2−^ on the growth and activity levels of fungi were subsequently tested to reflect their ROS sensitivity. The growth of the Δ*Asp f3* strain decreased sharply after treatment with xanthine oxidase and xanthine, an enzymatic reaction that produces free radicals, indicating that the Asp f3 deletion mutant is sensitive to ROS [58]. In addition, spot assays of H_2_O_2_ sensitivity in *prx* single and double mutants showed that the mutants exhibited higher sensitivity compared with the WT cells of *S. cerevisiae* [61]. In this study, the inactivation of *Aoprdx2* led to high sensitivity to H_2_O_2_ and menadione, and the mycelia of the ∆*Aoprdx2* mutant accumulated more ROS. Therefore, Prx homologs exhibit conserved functions in cellular antioxidant defenses in *A. oligospora* and other fungi.

NT fungi can produce a wide range of metabolites during mycelial growth and in relation to trophic transition [62,63]. Fungal cells employ various metabolism-related effectors or mechanisms in scavenging ROS, such as the induction of NADPH fluxes by switching to the pentose phosphate pathway [64], as well as the production of a number of secondary metabolites, such as aflatoxins, gliadin, and ochratoxins [65,66]. Similarly, in many filamentous fungi, Prx regulates a variety of metabolic processes and is associated with ROS detoxification. In *A. nidulans*, in addition to the antioxidant response and developmental defects, Δ*prxA* and Δ*prxB* mutant strains grow very poorly in a medium containing ethanol, arabinose, or fructose as the sole carbon source [31]. Genome-wide transcription studies of *Cryptococcus neoformans* showed that Tsa1 affects cell differentiation, melanin production, and resistance to azole antifungals. Fluoxonitrile mutants showed increased melanin production and resistance to antifungals [67]. Here, the deletion of *Aoprdx2* resulted in a severe reduction in the abundance of metabolites, such as arthrobotrisins. Previous studies have shown that arthrobotrisins are involved in the regulation of mycelial growth and trap formation in *A. oligospora* and other NT fungi [68]. In this study, the inactivation of *Aoprdx2* resulted in a reduction in the biosynthesis of arthrobotrisins and trap formation. In addition, in the metabolic pathway, a lot of cholesterol biosynthesis and fatty acid biosynthesis compounds are enriched, indicating that Prx further regulates various cellular processes by regulating different metabolic pathways (Table S3). Therefore, Prx homologs play multiple roles in secondary metabolism.

## 5. Conclusions

This study demonstrated that AoPrdx2 is a conserved regulator involved in multiple cellular processes, especially in oxidative stress, and is essential for the regulation of ROS, spore production, and secondary metabolism. AoPrdx2 plays a role in nucleus and septum development and endocytosis. Importantly, AoPrdx2 regulates trap formation and exerts a key role in nematode predation efficiency. Taken together, this study revealed, for the first time, the pleiotropic roles of the Prx family protein in NT fungi. This study establishes a connection between oxidative stress and trap formation and contributes to elucidating the mechanism involved in the lifestyle transition of NT fungi.

## Data Availability

Data are contained within the article and supplementary materials.

## Supplementary Materials

The following supporting information can be downloaded at: https://www.mdpi.com/article/10.3390/jof10020110/s1. Figure S1: Multiple sequence alignment and phylogenetic analysis of Prdx2 homologs from different fungi; Figure S2: Knockout and validation of the *Aoprdx2* gene; Figure S3: Comparison of stress response to cell-wall-disturbing reagents and hyperosmotic reagents; Table S1: Primers used for gene knockout in this study; Table S2: Dry weights of the wild-type (WT) and mutant strains before sample loading and the volume of methanol at a chromatographic level; and Table S3: The top 50 pathways associated with differentially expressed compounds.
