# Supplementary material for: AoPrdx2 Regulates Oxidative Stress, Reactive Oxygen Species, Trap Formation, and Secondary Metabolism in Arthrobotrys oligospora
Source: J Fungi (Basel). 2024 Jan 28;10(2):110. doi: 10.3390/jof10020110 (PMC10890406; doi:10.3390/jof10020110)
Supplement: Supplementary file 1 [file jof-10-00110-s001.zip › jof-2804841-supplementary.pdf]

## Supporting Information

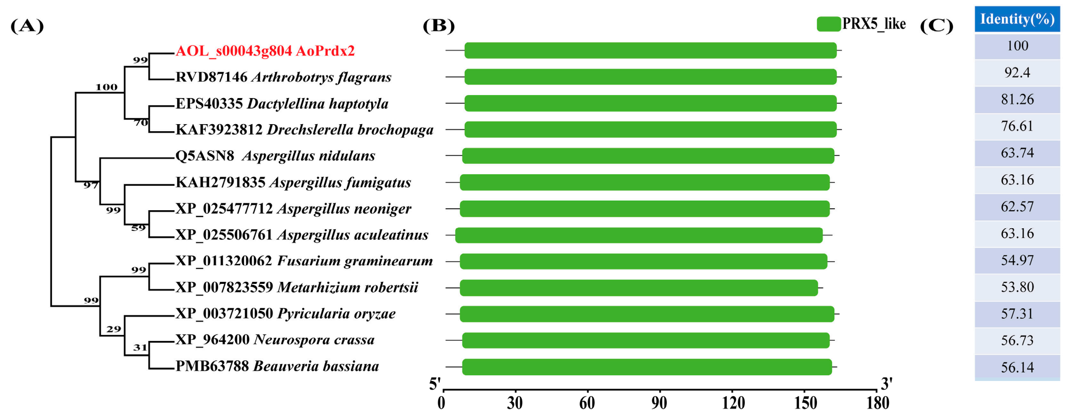


**Figure S1**. Multiple sequence alignment and phylogenetic analysis of Prdx2 homologs from different fungi. (A) Phylogenetic tree of Prdx2 homologs from different fungi. The GenBank number of Prdx2 homologs is shown. (B) Analysis of conserved domain (color region) of the Prdx2 homologs. (C) Comparison of sequence similarity between AoPrdx2 and other homologs from different filamentous fungi.

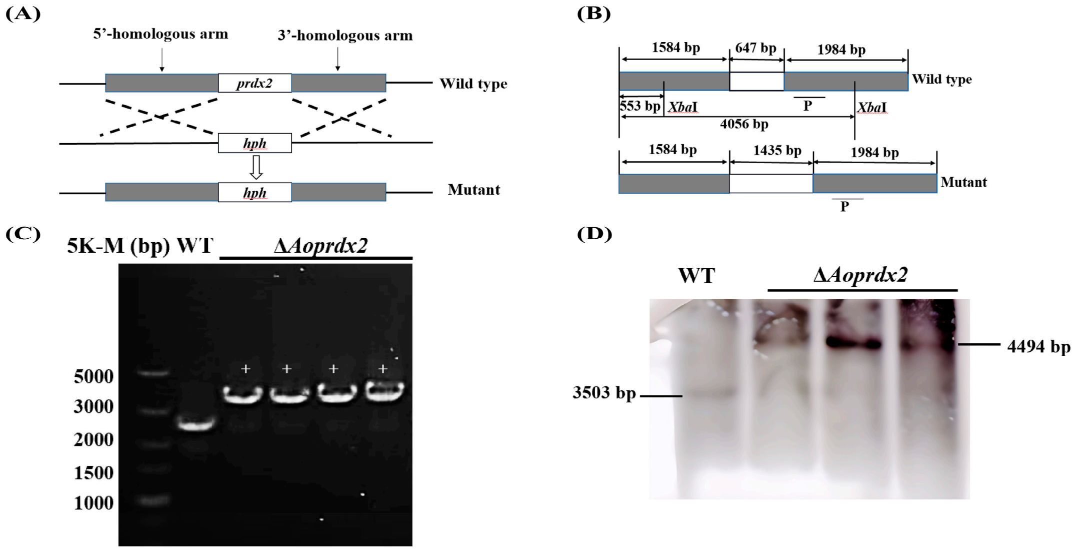


**Figure S2**. Knockout and validation of the *Aoprdx2* gene. (A) Diagram of gene knockout via homologous recombination. (B) Diagram of homologous sequences of target gene, *hph* fragment, Southern blotting probe (p), and restriction endonuclease sites (*Xba*I). (C) Verification of positive transformants by PCR amplification. 5K-M (bp), DNA Marker; WT indicates the wild-type strain; Δ*Aoprdx2* indicates the mutant strains. (D) Verification of three Δ*Aoprdx2* mutants by Southern blotting analysis.

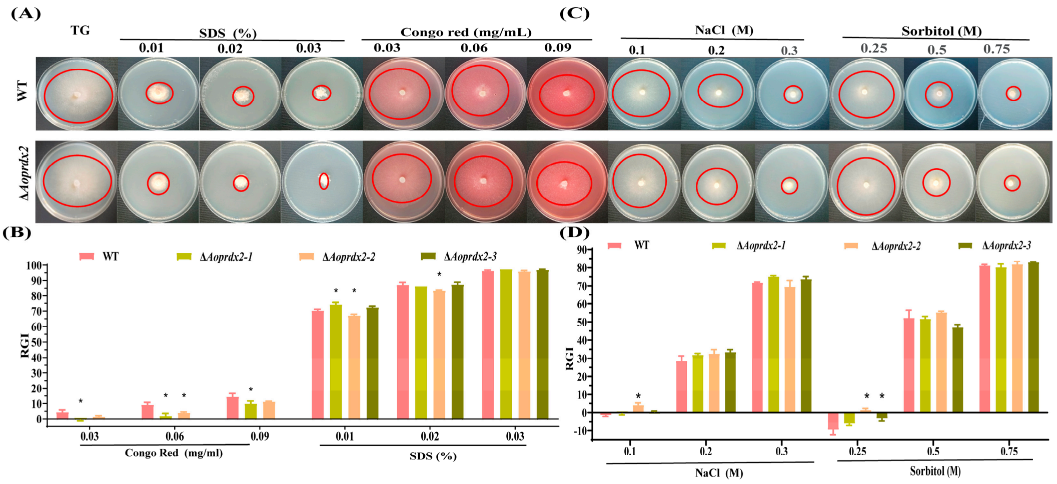


**Figure S3**. Comparison of stress response to cell-wall-disturbing reagents and hyperosmotic reagents. (A) Colony morphology of the WT and Δ*Aoprdx2* mutant strains on TG medium supplemented with different concentration gradients of cell-wall-disturbing reagents. (B) Relative growth inhibition rate (RGI) of the WT and Δ*Aoprdx2* mutant strains under stresses of cell-wall-disturbing reagents. (C) Colony morphology of the WT and Δ*Aoprdx2* mutant strains on TG medium supplemented with different concentration gradients of hyperosmotic reagents. (B) RGI values of the WT and Δ*Aoprdx2* mutant strains under stresses of hyperosmotic reagents. Indicates that the Δ*Aoprdx2* mutant strain is significantly different from the WT strain (Tukey's HSD, $p < 0.05$).

**Table S1**. Primers used for gene knockout in this study.

| Primers | Sequence (5′-3′) | Description |
|---|---|---|
| Aoprdx2-5f | GTAACGCCAGGGTTTTCCCAGTCACGACGAATCTATCGCTTCTGTCTT | Amplify the *Aoprdx2* gene 5’ flank |
| Aoprdx2-5r | ATCCACTTAACGTTACTGAAATCTCCAACAACCGAACTGAATAACCT | |
| Aoprdx2-3f | CTCCTTCAATATCATCTTCTGTCTCCGACGCGAGGTTACTGTTAGCG | Amplify the *Aoprdx2* gene 3’ flank |
| Aoprdx2-3r | GCGGATAACAATTTCACACAGGAAACAGCTCAACTTTCCAAGCGTCT | |
| hphF | GTCGGAGACAGAAGATGATATTGAAGGAGC | Amplify the *hph* cassette |
| hphR | GTCGGAGACAGAAGATGATATTGAAGGAGC | |
| yz-5f | AAGCCCGAGCATAGTCAG | Verify the transformants via PCR amplification |
| yz-3r | TTGGTCGTTTATCCGAAG | |
| tz-5f | GTACACGAATAACCCACTC | Make Southern blotting probe |
| tz-3r | TAATAACAACCACCTCCC | |

Table S2. Dry weights of the wild-type (WT) and mutant strains before sample loading and volume of methanol at chromatographic level.

| **Samples** | **Mycelial dry weight (g)** | **Chromatography-grade methanol (mL)** | **Final concentration (mg/mL)** |
|---|---|---|---|
| WT_1 | 0.82 | 820 | 10 |
| WT_2 | 0.86 | 860 | 10 |
| WT_3 | 0.87 | 870 | 10 |
| Δ*Aoprdx2*_1 | 0.90 | 900 | 10 |

| | | | |
|---|---|---|---|
| Δ*Aoprdx2*_2 | 0.87 | 870 | 10 |
| Δ*Aoprdx2*_3 | 0.89 | 890 | 10 |

Table S3. The top 50 pathways associated with differentially expressed compounds.

| **KEGG pathways** | **Compounds in Pathway** |
|---|---|
| Superpathway of cholesterol biosynthesis | 168 |
| Superpathway of chorismate metabolism | 152 |
| Superpathway of fatty acids biosynthesis | 149 |
| Superpathway of histidine, purine, and pyrimidine biosynthesis | 149 |
| Superpathway of flavones and derivatives biosynthesis | 147 |
| Methanobacterium thermoautotrophicum biosynthetic metabolism | 139 |
| Superpathway of gibberellin biosynthesis | 135 |
| Superpathway of fatty acid biosynthesis II (plant) | 127 |
| Plant sterol biosynthesis | 117 |
| Superpathway of aromatic compound degradation via 2-oxopent-4-enoate | 102 |
| Adenosylcobalamin biosynthesis II (aerobic) | 101 |
| Adenosylcobalamin biosynthesis I (anaerobic) | 99 |
| Superpathway of ergosterol biosynthesis I | 98 |
| Superpathway of L-lysine degradation | 97 |
| Superpathway of C28 brassinosteroid biosynthesis | 97 |
| Superpathway of cholesterol degradation II (cholesterol dehydrogenase) | 95 |
| Superpathway of diterpene resin acids biosynthesis | 93 |
| Superpathway of purine nucleotides de novo biosynthesis II | 86 |
| Superpathway of trichothecene biosynthesis | 84 |
| Superpathway of aerobic toluene degradation | 82 |
| Superpathway of aflatoxin biosynthesis | 80 |
| Superpathway of steroid hormone biosynthesis | 79 |
| Superpathway of pentose and pentitol degradation | 79 |
| Superpathway of cholesterol degradation I (cholesterol oxidase) | 78 |
| Superpathway polymethylated quercetinquercetagetin glucoside biosynthesis | 78 |
| Superpathway of aromatic compound degradation via 3-oxoadipate | 77 |
| Superpathway of bacteriochlorophyll a biosynthesis | 75 |
| Cholesterol biosynthesis I | 75 |
| Cholesterol biosynthesis III (via desmosterol) | 75 |
| Cholesterol biosynthesis II (via 24,25-dihydrolanosterol) | 75 |
| Anaerobic aromatic compound degradation (Thauera aromatica) | 74 |
| Superpathway of mycolyl-arabinogalactan-peptidoglycan complex biosynthesis | 74 |
| Aspartate superpathway | 74 |
| Superpathway of purine nucleotides de novo biosynthesis I | 74 |
| Novobiocin biosynthesis | 60 |
| Superpathway of isoflavonoids (via naringenin) | 60 |
| Superpathway of megalomicin A biosynthesis | 60 |
| Superpathway of microbial D-galacturonate and D-glucuronate degradation | 60 |

| | |
|---|---|
| Superpathway of betalain biosynthesis | 59 |
| Superpathway of glycolysis, pyruvate dehydrogenase, TCA, and glyoxylate bypass | 59 |
| Superpathway of methanogenesis | 58 |
| Kanamycin biosynthesis | 57 |
| Superpathway of erythromycin biosynthesis | 57 |
| Superpathway of rifamycin B biosynthesis | 57 |
| Superpathway of pyrimidine deoxyribonucleotides de novo biosynthesis | 56 |
| Superpathway of dTDP-glucose-derived antibiotic building blocks biosynthesis | 56 |
| Superpathway of cytosolic glycolysis (plants), pyruvate dehydrogenase and TCA cycle | 55 |
| Peptidoglycan biosynthesis II (staphylococci) | 55 |
| Peptidoglycan biosynthesis IV (Enterococcus faecium) | 55 |
| Superpathway of purine nucleotide salvage | 54 |
| Anaerobic aromatic compound degradation (Thauera aromatica) | 74 |
| Superpathway of mycolyl-arabinogalactan-peptidoglycan complex biosynthesis | 74 |
| Aspartate superpathway | 74 |
| Superpathway of purine nucleotides de novo biosynthesis I | 74 |
| Novobiocin biosynthesis | 60 |
| Superpathway of isoflavonoids (via naringenin) | 60 |
| Superpathway of megalomicin A biosynthesis | 60 |
| Superpathway of microbial D-galacturonate and D-glucuronate degradation | 60 |
| Superpathway of betalain biosynthesis | 59 |
| Superpathway of glycolysis, pyruvate dehydrogenase, TCA, and glyoxylate bypass | 59 |
| Superpathway of methanogenesis | 58 |
| Kanamycin biosynthesis | 57 |
| Superpathway of erythromycin biosynthesis | 57 |
| Superpathway of rifamycin B biosynthesis | 57 |
| Superpathway of pyrimidine deoxyribonucleotides de novo biosynthesis | 56 |
| Superpathway of dTDP-glucose-derived antibiotic building blocks biosynthesis | 56 |
| Superpathway of cytosolic glycolysis (plants), pyruvate dehydrogenase and TCA cycle | 55 |
| Peptidoglycan biosynthesis II (staphylococci) | 55 |
| Peptidoglycan biosynthesis IV (Enterococcus faecium) | 55 |
| Superpathway of purine nucleotide salvage | 54 |
